# Diffusion, convergence and influence of pharmaceutical innovations: a comparative study of Chinese and U.S. patents

**DOI:** 10.1186/s12992-018-0408-z

**Published:** 2018-08-30

**Authors:** Qiaolei Jiang, Chunjuan Luan

**Affiliations:** Dalian University of Technology, No. 2 Linggong Road, Ganjingzi District, Dalian, 116024 Liaoning Province China

**Keywords:** The pharmaceutical industry, Diffusion of innovation, China, U.S, Technology convergence, Network analysis

## Abstract

**Background:**

Despite the significant impact of pharmaceutical innovations on healthcare, our understanding is still limited because previous studies explored only a few cases and largely came from a linear perspective. This study presents a detailed case of the Chinese and U.S. pharmaceutical patents and investigated advancements that the global pharmaceutical industry is experiencing. A network analysis approach was used to identify certain aspects regarding the diffusion of pharmaceutical innovations, including innovation attributes, adopter characteristics, and clustering.

**Methods:**

Based on a patent database, network analysis and visualization, this study captured the structure of patent networks for the global pharmaceutical landscape in a large set of patents. A large volume of patent data, 15,422 patent filings citing Chinese pharmaceutical patents, 28,075 citing U.S. patents, and 6064 citing both Chinese and U.S. patents during 2014–2015, were retrieved from the world patent database, *Derwent Innovation Index*. The networks reveal many interesting features of technological innovation, convergence trends and diffusion patterns.

**Results:**

Convergence innovations were identified, with the advantage and influence of U.S. patents shown in a variety of areas, and their Chinese counterparts were concentrated in traditional Chinese medicine. Early adopters of Chinese patents were mainly universities within the national sector, while early adopters of U.S. patents were academic institutions and large international pharmaceutical corporations of balanced quantity, contributing a higher degree of technology convergence. Technology convergence in the cancer-treatment sector is expected to have a high future development potential.

**Conclusion:**

Chinese and U.S. pharmaceutical innovations contributed differently to the growth and development of the global pharmaceutical industry. The findings of this study can provide rich knowledge about the influence, diffusion and convergence trends of Chinese and U.S. pharmaceutical innovations. In the pharmaceutical industry, the findings may provide implications for researchers, policy makers, health professionals, and the general public to help improve the overall health of society.

## Background

Different from many other industries, in the pharmaceutical industry, most expenses are not in the manufacturing of goods but rather in the process of researching and developing safe, effective and marketable drug products, which is very costly and time-consuming [1, 2]. The success of the pharmaceutical industry relies heavily on innovations made during research and development (R&D), especially in the form of patents [1, 3, 4]. The prominent role played by patents within the pharmaceutical domain is unquestionable and provides new opportunities for pharmaceutical companies to expand and build new technologies, corporate capabilities, and market competitiveness [5–9].

The severity and gravity of global and local public health needs compel urgent scrutiny of the pharmaceutical patent system, which has now become global [10]. For a long time, the U.S. constituted the only hub in the network of pharmaceutical innovations [11, 12]. The U.S. pharmaceutical industry is strongly committed to R&D, especially in the patenting of inventions concerned with biotechnological aspects of therapy and diagnosis [11, 13, 14]. With more than 18% of the world’s population, China only accounts for a small percentage of the global pharmaceutical industry [1, 15]. The Chinese government has been making great efforts to boost pharmaceutical development and has achieved noticeable results given its long and rich pharmaceutical history, skillful workforce, cost-effective resources and unmatched potential domestic market size [1, 14, 16]. Insufficient intellectual property rights protection in China is a major concern for many pharmaceutical companies, while over the years, China has devoted considerable efforts to bring its patent practice in line with accepted international standards, with local patent applications stably increasing [1, 17–19]. In recent years, the apparent productivity crisis in the global pharmaceutical industry and the economic and political rise of China have contributed to renewed interest in the application of Chinese medicine for drug discovery [20]. Composed of a number of treatment modalities, most notably herbal medicines and acupuncture, Traditional Chinese medicine (TCM) has been found to have a large advantage in many disease clinical treatments, owing to its low toxicity and side-effects relative to western medicine [21, 22]. TCM has gained increasing popularity as alternative medicines in the developed world, as well as in the Third World, where limited resources are available for medical care [21]. During the Nobel Lectures in Physiology or Medicine, China’s pharmacologist Youyou Tu made a keynote speech entitled *Discovery of Artemisinin – a gift from TCM to the world*, in which she compared Chinese medicine and pharmacology to a great treasure-house and highlighted that more attention should be paid to TCM research and its potential for discoveries of more novel medicines beneficial to world healthcare [23–28].

The pharmaceutical sector is in a process of innovation convergence, with the spread of the patent system building innovation economies that would not exist otherwise. The objective of this paper is to capture this influence of patent-induced diffusion of innovations on the dynamics of R&D and industry development, with a comparative study of Chinese and U.S. pharmaceutical patents.

## Methods

### Theoretical framework

Our methodological approach is informed by the literature on the Diffusion of Innovations (DOI) theory and technology convergence literature. Therefore, we review Diffusion of Innovations (DOI) theory and the technology convergence literature, which guide the subsequent analyses of trends and patterns in the global pharmaceutical industry.

Given the substantial amount of time and money invested in innovative new drugs and medical innovations, diffusion of innovation is crucial for development of the pharmaceutical industry and availability of affordable medicines for healthcare [4, 29]. With a broad scope of practical applications in the fields of medicine, public health, and health communication, DOI is one of the classic approaches to study diffusion, as the process through which an innovation spreads via certain communication channels over time among the members of a social system [30–37]. The pattern of international patenting reflects the channel between the source and the destination of transferred technology [38]. Hence, patent data have been studied as indicators of DOI [38–40]. Although the pharmaceutical industry has become increasingly global, most of the studies examined the growth in patent filings in individual countries, without focus on the world as a whole [41]. This study sought to fill this gap by conducting an analysis of global patenting trends using comprehensive archival data over time. Based on DOI theory, innovation attributes, adopter characteristics, and clustering were used to achieve the objective of exploring and depicting diffusion patterns of pharmaceutical innovations.

**Fig. 1:**
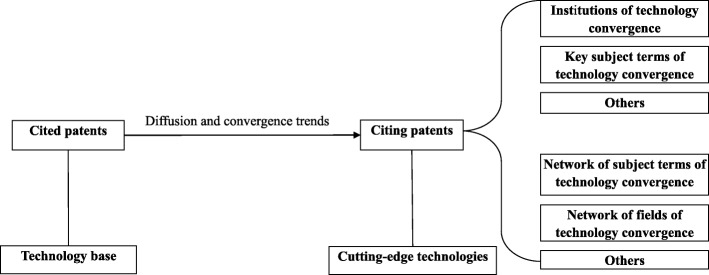
Conceptual framework

Technology convergence is such a convergence innovation that its process can be featured as a learning process that is a continuous disequilibrium between reference technology and its matching technology, which adjusts the optimal balance between the functions of the two technologies [42–44]. Currently, technology convergence-inducing innovations are a major driver of development [45]. Technology convergence is being witnessed in various sectors and most likely occurs in those areas that still have patentable inventions, such as the pharmaceutical industry [46–49]. Convergence and re-orientation processes within the pharmaceutical sector are actively progressing and embody many different types of novel combinations representing syntheses of therapeutic, diagnostic and digital information technologies, as well as new drug R&D, such as DNA diagnostics, neural computation, molecular electronics and bioinformatics [43, 44, 50, 51]. Empirically, the network analysis perspective has been widely used to investigate technology convergence [12, 42, 49, 52–59]. The structure of technological interrelationship of patent networks was analyzed to capture the technology convergence patterns and to identify possible future cutting-edge frontiers in the global pharmaceutical industry [54, 58, 60–65]. The conceptual framework is presented in Fig. 1.

### Data sources and selection

As one of the most comprehensive databases collecting patent documents in the world, *Derwent Innovation Index* (*DII*) was used for data retrieval. In this study, the technology classification code “B”, which refers to “Pharmaceuticals” in *DII*, was chosen for data retrieval. We searched the patent publications during the time span of 2014–2015, specifically those citing pharmaceutical patents granted by the State Intellectual Property Office of P.R.C (Chinese patents), pharmaceutical patents granted by the U.S. Patent and Trademark Office (U.S. patents), and both. In this study, “China” refers to Mainland China. In total, 15,422 patent filings citing Chinese pharmaceutical patents, 28,075 patent filings citing U.S. patents, and 6064 citing both were retrieved and downloaded during February 6–10, 2016. The data include patent number, title, author names (inventor names), patent assignee name(s) and code(s), Derwent primary accession number, abstract, Derwent class code, international patent classification, patent publication date, application details and date, priority application information and date, cited patents, etc.

### Network analysis

A network analysis approach was used to explore the development of technology diffusion, convergence and influence. As the conceptual framework presents in Fig. 1, to investigate and compare technology convergence from China and U.S. in the global pharmaceutical industry, we studied the clustering of patent data citing patents from China and U.S., respectively and jointly.

### Network visualization

Excel, CiteSpace [66, 67], Bibexcel [68], and Ucinet [69] software programs were employed to analyze the patent data. CiteSpace [70, 71] was used to conduct co-occurrence analysis on topic terms from citing patent titles, which reflect the nature, characteristics and novelty of patent technology precisely after being indexed by *DII* [72]. NetDraw, as a network visualization program embedded in Ucinet, was employed to generate network graphs based on the net documents generated by CiteSpace [73, 74, 75].

## Results

### Influential innovations in the global pharmaceutical patent landscape

In this study, the cited pharmaceutical patents are regarded as innovations with the patent holders as innovators, and the assignees can be seen as early adopters. As aforementioned, 28,075 patent filings citing the pharmaceutical innovations patented in the United States and 15,422 citing Chinese pharmaceutical patents were retrieved. The United States still plays a more influential role in the global pharmaceutical industry in general compared with the situation of previous timeframes, for example, during 1994–1995 there were 18,328 citing U.S. patents and only 66 citing Chinese patents. However, it is worth noting that in a relatively short time Chinese pharmaceutical patents are becoming more visible and influential in the global pharmaceutical patent landscape with a great amount of increase.

To determine the influential innovations in the global pharmaceutical industry, frequency analyses were conducted to examine the subject terms extracted from the titles, which showcase the main characteristics and innovative aspects of the patents [76]. The most frequently cited subject terms, or converging subject terms, illustrate the currently influential innovations in the global pharmaceutical industry. According to the results (see Table 1), for Chinese pharmaceutical innovations, the most frequently cited subject term was traditional Chinese medicine (TCM). Among the top 10 converging subject terms, seven were related to TCM. While the influences of U.S. pharmaceutical innovations are more dispersed. The top 10 converging subject terms included pharmaceutical composition, treating cancer, biological sample, rheumatoid arthritis, Alzheimer’s disease, nucleic acid, and multiple sclerosis. The results are presented in Table 1.

**Table 1:** Top 10 converging subject terms in the global pharmaceutical industry (China vs. the United States)

Ranking	Subject term (China)	Record count	Subject term (U.S.)	Record count
1	traditional Chinese medicinal composition	673	pharmaceutical composition	1049
2	traditional Chinese medicine	589	treating cancer	590
3	pharmaceutical composition	440	breast cancer	287
4	radix astragali	245	biological sample	286
5	preparing medicine	233	rheumatoid arthritis	280
6	orange peel	212	alzheimers disease	266
7	radix codonopsitis	186	nucleic acid	238
8	rhizomeligusticiwallichii	172	multiple sclerosis	205
9	rhizomecoptidis	159	treating disease	194
10	treating cancer	156	lung cancer	187

### Early adopters of innovations in the global pharmaceutical industry

To determine the early adopters of innovations in the global pharmaceutical industry, frequency analyses were conducted regarding the assignee names of the patent filings. The results (see Table 2) showed that during 2014–2015, the top 10 assignees most frequently citing Chinese pharmaceutical patents were all Chinese institutions. The influence of Chinese pharmaceutical innovations seemed to be more within the national level than moving to the international or global level. Among the ten top-ranked converging assignees, six were Chinese universities, and only two were Chinese pharmaceutical corporations. As early adopters, these top 10 Chinese institutions all contributed less than 1%, and together only 3.84% of the 15,422 patents retrieved, which showed a relatively lower degree of convergence.

**Table 2:** Top 10 converging assignees in the global pharmaceutical industry (China vs. the United States)

Ranking	Assignee name (China)	% of 15,422	Assignee name (U.S.)	% of 28,075
1	Zhejiang University	0.73	F. Hoffmann-La Roche Ltd.	1.75
2	Jiangnan University	0.53	University of California	1.03
3	Shanghai Jiao Tong University	0.37	Merck Sharp & Dohme Corp.	0.88
4	Shanghai Institute of Pharmaceutical Industry	0.34	Inserm Inst Nat Sante & Rech Medicale	0.53
5	China Pharmaceutical University	0.33	Novartis AG	0.50
6	Shandong New Hope LIUHE Group Co. Ltd.	0.32	Sanofi-Aventis Deutschland GmbH	0.49
7	Jinan University	0.32	Harvard College	0.47
8	Nanjing Guangkangxie BioPharma Co. Ltd.	0.32	University of Texas System	0.47
9	Qingdao Municipal Hospital	0.31	Roche Diagnostics GmbH	0.46
10	Shandong University	0.27	Johns Hopkins University	0.45

For the top 10 assignees most frequently citing U.S. pharmaceutical patents, large conglomerates were the major contributors. Although universities also account for a significant proportion of the top 10, we found more international or transnational corporations. The degree of convergence was higher than their Chinese counterparts, with the ten top-ranked institutions accounting for 7.03% of the patent filings citing U.S. pharmaceutical patents. For example, as the most active early adopter, F. Hoffmann-La Roche accounted for 1.75% of the total 28,075 global patent filings citing U.S. pharmaceutical innovations. The results are presented in Table 2.

### Frontiers of the global pharmaceutical industry based on convergence innovations

The high-frequency subject terms extracted from cited patents indicate possible cutting-edge frontiers toward which the industry is advancing. By employing CiteSpace, we extracted 256 subject terms from the titles of the patent filings citing Chinese pharmaceutical patents, 274 subject terms from the patent data citing U.S. patents, and 147 from the patent filings citing both. The visualized convergence maps were generated by using NetDraw. The clusters were labeled with consideration of the meaning of the subject terms, as denoted by nodes in the networks.

**Fig. 2:**
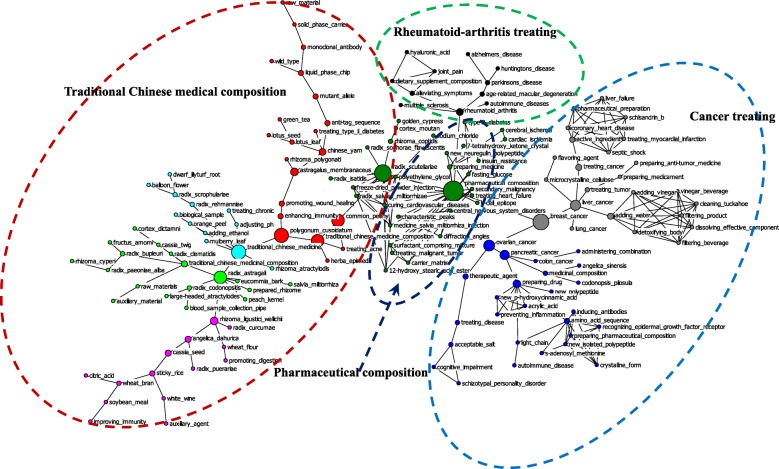
Convergence of Chinese pharmaceutical innovations in the global pharmaceutical industry

Based on the results (see Fig. 2), four convergence innovation sub-networks were shown by those citing Chinese pharmaceutical patents. The largest one was “traditional Chinese medical composition”, within which most of the subject terms were Chinese herbal medicines or TCM compositions. The following convergence innovation cluster was “cancer treatment”, within which some terms played significant mediating roles, such as breast cancer, ovarian cancer, liver cancer, and pancreatic cancer. The third convergence innovation sub-network was “pharmaceutical composition”, within which pharmaceutical composition played crucial mediating effects. The fourth convergence innovation sub-network was “rheumatoid arthritis treatment”, and the term rheumatoid arthritis played an important mediating role.

**Fig. 3:**
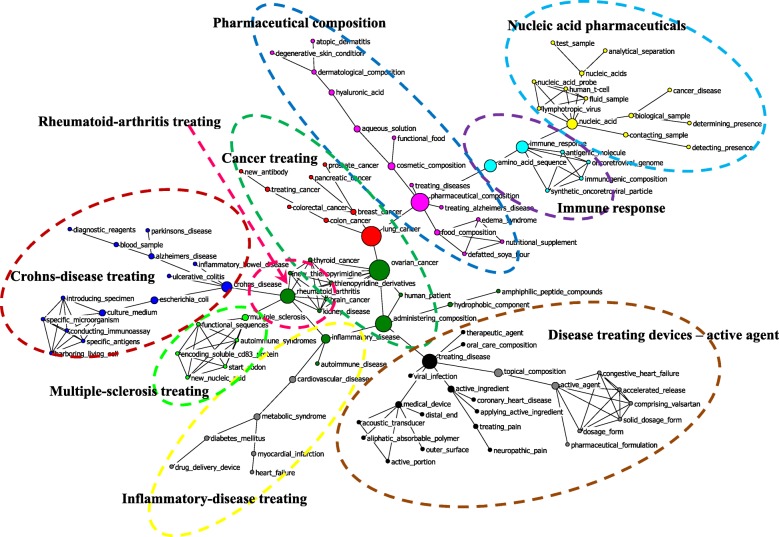
Convergence of U.S. pharmaceutical innovations in the global pharmaceutical industry

Comparatively, more new frontiers were opening up with the convergence innovations citing U.S. pharmaceutical patents, in total nine such convergence innovations (see Fig. 3). Among them, one key convergence innovation was “cancer treatment”, within which the following terms played significant mediating roles, including ovarian cancer, lung cancer, and administering composition. New antibodies made up an emerging research subject in this convergence innovation. Another key convergence innovation was “disease treating devices – active agent”, within which the following terms played important mediating functions: treating disease, topical composition, active agent, etc. “Pharmaceutical composition” was also a main convergence innovation, within which some terms played crucial mediating effects, including pharmaceutical composition, cosmetic composition, and aqueous solution. Shown in the top right corner, “nucleic acid pharmaceuticals” was also an influential convergence innovation. Moreover, the convergence innovations citing U.S. pharmaceutical patents also included Crohn’s-disease treatment, inflammatory-disease treatment, multiple-sclerosis treatment, and immune response and rheumatoid-arthritis treatment.

**Fig. 4:**
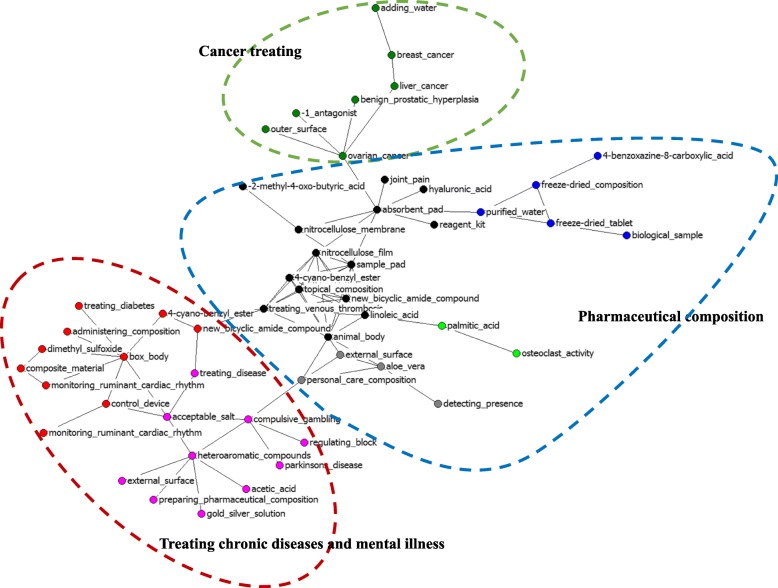
Convergence of both Chinese and U.S. pharmaceutical innovations in the global pharmaceutical industry

Regarding the frontiers of the global pharmaceutical industry based on convergence innovations citing both Chinese and U.S. patents, three main convergence innovations were found (see Fig. 4). The largest frontier was “pharmaceutical composition”, followed by “cancer treating” and “treating chronic diseases and mental illness.” Within the convergence innovation of “pharmaceutical composition”, there were some terms that played important mediating functions, such as freeze-dried composition, freeze-dried tablets, sample pads, topical composition, and nitrocellulose film. Within “cancer treatment” cluster, the following terms played significant mediating roles, including breast cancer and liver cancer. Preparing pharmaceutical composition, acceptable salt, and treating disease among others showed important mediating functions in the convergence of “treating chronic diseases and mental illness”.

## Discussion

Based on patent data, this study examined and compared the developing trends influenced by Chinese and U.S. pharmaceutical innovations by employing network analysis and visualization techniques. The implications for growth and technological evolution of the global pharmaceutical industry were discussed.

The patent data reflect that the Chinese patent system is growing rapidly, and at a pace faster than its U.S. counterpart. Empirical results revealed that U.S. pharmaceutical innovations showed their advantage and influence in a variety of technological fields, while China significantly contributed to the growth and industrial development of TCM. The Chinese patent system is fostering new industries, which leverage TCM in ways that build economies and improve human health by paying more attention to TCM research and its potential for discoveries of more novel medicines beneficial to world healthcare, for example, new cancer medications. In recent years, the development of TCM has been legally and officially ensured with certain policies, such as the 13th Five-Year Plan and the publication of the *Law of the People’s Republic of China on Traditional Chinese Medicine* in 2017. Accordingly, more and more effective therapeutic components of TCM have been patented, and combinations of synthetic compounds and TCM emerged as a novel trend for the modernization of TCM. Given the competitive advantages of TCM, development strategies for the Chinese pharmaceutical industry are suggested to consolidate local and global markets for herbal and generic drugs [15]. The Chinese public entities, such as public universities, research institutions and public hospitals, have recognized and are attempting to capitalize on the pharmacologic and commercial potential of TCM, which has also been officially supported. Currently, the public entities have managed to steer the direction of innovation in China. It can be a good thing for public health, since the government-supported public entities play an active role not only in the innovation and development of TCM but also in controlling price and other procedures beneficial for public health. Meanwhile, the Chinese government is also trying to boost morale of private and even overseas pharmacological ventures and practitioners to have more patents with significant impacts on the development of TCM all over the world.

According to the results, the benefits of strengthening patent laws are more realized by multi-national firms, especially with the current situation of the United States continuing to dominate the pharmaceutical patent landscape. Large and multinational pharmaceutical companies can usually better acquire technological capabilities and innovative competencies in the pharmaceutical sector [77, 78], with greater and more international influence. Differently, the influence of Chinese pharmaceutical innovations was more within the national sector than international sectors of the industry. More than half of the early adopters of Chinese pharmaceutical innovations were Chinese universities, with a limited presence of research-driven pharmaceutical companies as technology application pioneers. As in many developing countries, public expenditures on R&D are invested in university research and public laboratories, while industrial R&D is most often lagging behind [76]. In the future, more university-corporate joint patents may be encouraged, and Chinese R&D-based, innovative pharmaceutical companies should embark on the path to international presence to achieve global competitiveness in the pharmaceutical industry.

## Conclusions

This study provides an overview of the current development of the global pharmaceutical patent landscape, with a comparative study of Chinese and U.S. pharmaceutical patents through the lenses of DOI approach and using network analysis and visualization techniques. Exploring the convergence of both Chinese and U.S. pharmaceutical innovations allowed us to discover the technological trajectories under the influence of two different and sometimes disparate models of medicine within the context of globalization of medical practice. The results suggest that although the U.S. still leads in pharmaceutical innovation with more patents, China has become more visible and influential with a rapid increase in pharmaceutical patents, especially in TCM development. Therefore, Chinese public entities with governmental support and more private entities, as well as multinational firms, may seize the opportunities and create their innovative competencies and technological capabilities accordingly.

The findings of the study can provide rich knowledge about the influence, diffusion and convergence trends of Chinese and U.S. pharmaceutical innovations, which are of great theoretical and practical significance, with the relevance of an evolutionary approach to the analysis of DOI and technology convergence on the one hand and some main implications for the present and future of the industry on the other. Using comprehensive archival patent data over time may help overcome the recall problem and individual-blame bias. Network analysis and visualization research techniques rather than the traditional linear model were proposed and applied as alternative methodological approaches, which may become future prospects for DOI studies. In the pharmaceutical industry, the findings may provide implications for researchers, policy makers, health professionals, and the general public to help them understand the current pharmaceutical patent landscape and its potential for discoveries of more novel medicines beneficial to worldwide healthcare.

In this study, we analyzed the trends of diffusion based on patent data in a two-year interval during 2014–2015. Future studies can analyze longer time periods or different time intervals. Specific innovations, instead of Chinese and U.S. pharmaceutical innovations in general, can be examined in further studies to capture the diffusion of a specific pharmaceutical innovation. More research is needed to analyze more types of data, such as academic publications, industrial reports, and R&D projects.
